# Epithelioid Angiosarcoma Presenting With Bilateral Hemothorax and Hemoptysis: A Case Report

**DOI:** 10.1155/crpu/6324954

**Published:** 2026-08-21

**Authors:** Simon Ståhl, Christina Triantafyllidou, Petros Effraimidis, Simone Ignatova, Johan Asplund, Erica Holmberg, Jonas Agholme

**Affiliations:** ^1^ Department of Internal Medicine, Section of Pulmonary Medicine, Vrinnevi Hospital, Norrköping, Sweden; ^2^ Department of Pathology, Linköping University, Linköping, Sweden, liu.se; ^3^ Department of Radiology, Vrinnevi Hospital, Norrköping, Sweden; ^4^ Department of Thoracic and Cardiovascular Surgery, Linköping and Department of Health, Medicine and Caring Sciences, Linköping University, Linköping, Sweden, liu.se; ^5^ Department of Internal Medicine and Department of Health, Medicine and Caring Sciences, Linköping University, Norrköping, Sweden, liu.se

**Keywords:** angiosarcoma, epithelioid angiosarcoma, hemoptysis, hemothorax, primary pulmonary angiosarcoma

## Abstract

We report a case of a 77‐year‐old man presenting with bilateral hemothorax, hemoptysis, and multiple bilateral pulmonary nodules and lung infiltrates. Histopathological and immunohistochemical analysis of a surgically resected nodule revealed epithelioid angiosarcoma, and the overall radiological and operative findings favored pulmonary origin. No other primary sites outside the thorax could be identified. The patient was planned for palliative chemotherapy, but his condition deteriorated, and he died before treatment could be initiated. Angiosarcoma is a rare and highly aggressive endothelial malignancy, most commonly affecting the skin and subcutaneous tissue of the head and neck. Primary pulmonary angiosarcoma is extremely uncommon; pulmonary involvement more often occurs through metastatic spread. When angiosarcoma involves the lungs as a primary neoplasm, it may present with multiple nodules, hemorrhagic lesions, or diffuse infiltrative patterns, frequently leading to hemoptysis and respiratory symptoms. Clinical course can be rapid, and prognosis is poor, particularly in advanced thoracic disease.

## 1. Introduction

Angiosarcoma is a rare, high‐grade endothelial malignancy of vascular or lymphatic origin. Clinical course is typically aggressive, and prognosis is poor. Primary involvement of the lungs is extremely rare, and current knowledge derives primarily from case reports and small series [1]. We describe a case of thoracic epithelioid angiosarcoma presenting with massive bilateral hemothorax and hemoptysis, with clinical, radiological, and operative findings favoring pulmonary origin.

## 2. Case Report

A 77‐year‐old man presented to the emergency department after a syncopal episode while using the toilet. His general condition was poor, and he had experienced pronounced fatigue recently. His medical history included a colectomy for ulcerative colitis 13 years earlier, endovascular aortic repair for an aortic aneurysm 4 years earlier, and Type II diabetes. His prescribed medications included low‐dose rivaroxaban (2.5 mg twice daily), acetylsalicylic acid, metformin, empagliflozin, paracetamol, pregabalin, and oxycodone. He had smoked for approximately 28 years but had quit over a decade earlier. For the past 1.5 years, he had experienced persistent lower back pain that was unresponsive to physiotherapy and multiple analgesics. Magnetic resonance imaging of the lower back performed 11 months earlier had shown only degenerative changes without malignant findings. He had been in recent contact with his primary care physician due to unintentional weight loss, dysphagia, and reduced appetite. During the months before admission, he had become increasingly bedridden due to general fatigue and worsening back pain. One week before presentation, he had noticed blood‐tinged sputum.

On admission, he was tired and mostly bedridden. Physical examination revealed a low blood pressure of 86/62 mmHg, pulse rate 67 beats per minute, and oxygen saturation of 92% on room air. Auscultation of the lungs revealed crackles in the right lung and diminished breath sounds at the bases of both lungs. The remainder of the physical examination was unremarkable. Laboratory tests revealed hemoglobin 68 g/L, elevated white blood cell count (14.5 × 10^9^/L), and a normal C‐reactive protein level (8 mg/L). Coagulation tests revealed no abnormality.

**Figure 1:**
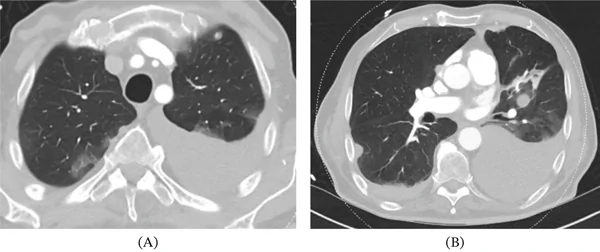
Initial chest computed tomography (CT), lung window. (A) Ground‐glass opacities in the upper lobes bilaterally, with a left‐sided pleural effusion. (B) Multiple pleural‐based nodules along the costal and mediastinal pleura in the right hemithorax and within the interlobar fissure of the left lung. A smaller right‐sided pleural effusion is also present.

A chest computed tomography (CT) scan with pulmonary angiography showed no evidence of pulmonary embolism but demonstrated bilateral pleural effusions, multiple bilateral nodular lesions located along the costal and mediastinal pleura and within the interlobar fissures, and bilateral ground‐glass opacities (Figure 1A,B).

The patient underwent ultrasound‐guided placement of pigtail drainage, through which several liters of grossly bloody fluid were sequentially drained from both pleural cavities. He required repeated erythrocyte transfusions to maintain an adequate hemoglobin level. Pleural fluid cultures were negative for bacteria, fungi, and mycobacteria. Cytological examination of both left‐ and right‐sided pleural fluid revealed no malignant cells. Pleural fluid hematocrit was not analyzed. However, at one point, pleural fluid hemoglobin from the right‐sided drainage was 70 g/L while blood hemoglobin was 85 g/L, and together with the grossly bloody pleural drainage, this supported the clinical interpretation of hemothorax.

**Figure 2:**
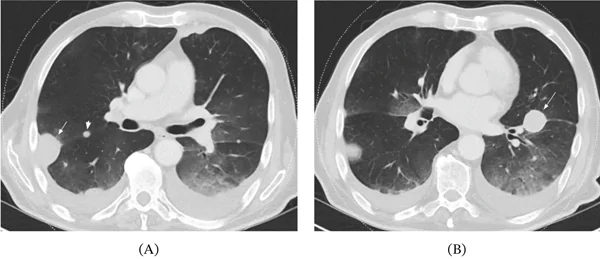
Chest computed tomography (CT) 6 days after admission. (A) Newly developed nodules in the right lung (arrowhead indicates one of the new nodules), together with progression of previously identified lesions (arrow). (B) Marked progression of a nodule along the left interlobar fissure (arrow). Small bilateral pleural effusions are also present.

Despite continuous drainage, the daily output of bloody fluid remained substantial, and the patient became dyspnoeic whenever attempts were made to reduce the drainage rate. He had a persistent cough with expectoration of fresh or coagulated blood. Repeated CT performed 6 days after admission showed newly developed nodules in the right lung and significant progression of the previously noted lesions (Figure 2A,B). Laboratory tests for autoimmune disease and vasculitis (antinuclear antibodies [ANAs], antineutrophil cytoplasmic antibodies [ANCAs], and antiglomerular basement membrane antibodies [anti‐GBMs]) and infectious diseases including HIV, hepatitis, and tuberculosis were negative. A lumbar CT scan, prompted by his chronic back pain, revealed no evidence of metastatic disease.

The patient was initially treated with broad‐spectrum antibiotics, which were discontinued when pleural fluid, blood, and sputum cultures were negative.

After multidisciplinary discussion, the patient underwent surgery with the initial intention of a video‐assisted thoracoscopic (VATS) biopsy of suspected right‐sided pleural nodules. Due to technical and anatomical considerations, including possible lung adhesions and the presence of hemothorax, the procedure was converted to a muscle‐sparing lateral minithoracotomy. The procedure was performed under general anesthesia with double‐lumen endotracheal intubation to allow single‐lung ventilation.

Upon entering the pleural cavity, a moderate amount of old blood and fluid was evacuated. The lung was adherent to the diaphragm. The visible parietal pleura was carefully inspected and appeared macroscopically normal. In contrast, the visceral pleura and lung surface appeared hemorrhagic and bled easily. Based on preoperative CT, a peripheral nodule adjacent to the right interlobar fissure was identified in the right upper lobe. Macroscopically, the nodule appeared to expand from the lung parenchyma, was poorly demarcated, and was hemorrhagic. A wedge resection including the nodule was performed. Because the disease already appeared widespread and the patient was frail, additional biopsies were not pursued after representative diagnostic tissue had been obtained. Concurrently, talc pleurodesis was performed.

**Figure 3:**
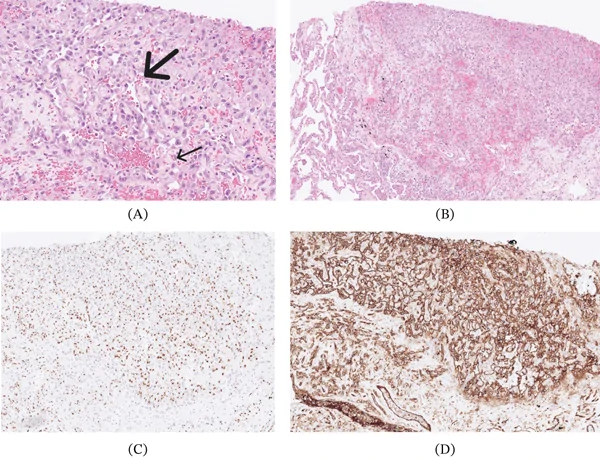
Histopathological findings. (A) ×40 magnification. Aggregates of epithelioid cells with pale cytoplasm and focal vasoformation (thick arrow). Some cells contain intracytoplasmic vacuoles with erythrocytes (thin arrow), representing primitive vascular lumina (“blister cells”). (B) ×20 magnification. Wedge resection specimen showing an infiltrative nodule of epithelioid angiosarcoma with associated hemorrhage and surrounding reactive lung parenchyma. (C) ×20 magnification. Immunohistochemical staining for ERG showing nuclear positivity in atypical cells. (D) ×20 magnification. Immunohistochemical staining for CD31 showing membranous staining in atypical cells.

Final histological analysis of the wedge resection confirmed epithelioid angiosarcoma. Parts of the specimen demonstrated reactive changes in pneumocytes, along with alveolar bleeding and the presence of hemosiderophages. Histologically, there were atypical cells—mainly epithelioid—with numerous mitoses. The epithelioid cells were arranged in small aggregates with focally suggested vasoformation, though without fully distinct or well‐defined vascular channels. The cells exhibited an epithelioid morphology with pale eosinophilic cytoplasm, vesicular nuclei with prominent nucleoli, and partially intracytoplasmic vacuoles representing primitive vascular lumina, commonly referred to as “blister cells.” Immunohistochemical staining was positive for CD31, ERG, CKAE1/AE3, and BAP1 and negative for calretinin, CD34, CD68, CK5, desmin, p40, SMA, SOX10, TTF‐1, and WT‐1, supporting the diagnosis of epithelioid angiosarcoma (Figure 3A–D).

**Figure 4:**
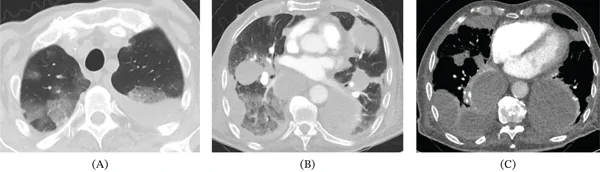
Chest computed tomography (CT) approximately 3 weeks after admission, after repeated bilateral drainage for hemothorax. (A) Worsening ground‐glass opacities in the upper lobes. (B) Marked and rapid progression of the pulmonary nodules, with a loculated pleural effusion on the left side and diffuse interstitial thickening in the right lower lobe. (C) Mediastinal window. Large, multiple, loculated hemorrhagic pleural effusions at both lung bases.

In consultation with oncology, the patient was planned for palliative systemic treatment. Because of his poor general condition (Performance Status 3) and advanced bilateral intrathoracic disease, a relatively less intensive monotherapy approach with weekly paclitaxel was chosen. However, his condition deteriorated successively; his respiratory symptoms intensified, and he required high oxygen flows to maintain adequate saturation. A new CT scan of the thorax revealed disease progression with relapse of bilateral pleural effusions (Figure 4A–C). Despite supportive care, his clinical condition continued to deteriorate, and he died 38 days after admission.

## 3. Clinical Timeline

Months before presentation, the patient experienced fatigue, lower back pain, weight loss, dysphagia, and reduced appetite. One week prior to presentation, he developed blood‐tinged sputum.

On Day 1, he presented to the emergency department, and rivaroxaban and acetylsalicylic acid were discontinued and not restarted. A left‐sided thoracic drainage was performed on Day 3. On Day 6, CT showed complete regression of the left‐sided pleural fluid and progression of the right‐sided pleural fluid, prompting right‐sided thoracic drainage on Day 7 due to relapse.

On Day 13, a decision was made to proceed with surgical biopsy, initially planned as VATS. On Day 17, the patient developed relapse of left‐sided hemothorax requiring a new left‐sided thoracic drainage. On Day 19, surgical biopsy was performed after conversion from VATS to a minithoracotomy, including wedge resection and talc pleurodesis.

Final histopathological diagnosis of epithelioid angiosarcoma was established on Day 34. On Day 36, the patient was planned for transfer to oncology for palliative chemotherapy. He died on Day 38.

## 4. Discussion

Angiosarcoma is a rare and aggressive form of soft tissue malignancy, accounting for approximately 2%–3% of all adult soft tissue sarcomas [2]. It most commonly involves the skin of the head and neck region or the breast, particularly after radiotherapy for breast cancer. Known risk factors include chronic lymphedema and prior radiotherapy [1]. Pulmonary involvement generally arises from metastatic spread originating in other primary sites [3].

Primary pulmonary angiosarcoma is extraordinarily rare. In a recent clinical analysis of 11 patients, the clinical presentation was nonspecific, and radiological findings included pulmonary nodules, pleural effusion, and ground‐glass opacities; prognosis remained poor, particularly in patients with multiple pulmonary lesions [4]. Its etiology remains unclear; however, several potential risk factors have been proposed, including tuberculosis‐related pyothorax [5], asbestos exposure [6], and prior radiotherapy [7]. Similar to previously reported pulmonary cases, our patient presented with hemoptysis, bilateral nodular lesions, and ground‐glass changes suggestive of alveolar bleeding [8–10].

Bleeding manifested as hemoptysis or hemothorax is a recurrent feature of thoracic angiosarcoma. Recent pulmonary and pleural case reports describe hemorrhagic presentations with ground‐glass opacities, pleural effusions, and rapid clinical deterioration [10–13]. However, the combined presentation of hemoptysis and hemothorax appears to be distinctly uncommon. In our focused literature review, we identified two clearly documented previous thoracic angiosarcoma cases reporting both findings [14, 15]. Compared with these reports, our case was unusual because of spontaneous bilateral hemothorax, bilateral pulmonary and pleural lesions, histological evidence of alveolar bleeding, and very rapid radiological progression within days.

An important diagnostic question in the present case is whether the tumor arose primarily from the lung or from the pleura. Primary pleural angiosarcoma is itself rare but is well known to present with hemothorax, pleural thickening, and pleural‐based masses [11–13]. In our case, the visible parietal pleura appeared macroscopically normal, whereas the visceral pleura and lung surface were hemorrhagic and bled easily. The biopsied nodule was located adjacent to the fissure and macroscopically appeared to expand from the lung parenchyma. In addition, the bilateral parenchymal nodules and ground‐glass opacities suggested diffuse alveolar bleeding rather than isolated pleural disease. For these reasons, we believe that the overall radiological, operative, and histopathological findings favored pulmonary origin, although a pleural origin cannot be excluded with absolute certainty.

Pathologic findings vary according to the degree of tumor differentiation. The growth pattern is usually infiltrative, lacking a distinct border with normal tissue. In well‐differentiated tumors, malignant cells form recognizable vascular structures, whereas in more aggressive tumors, the tissue architecture is disorganized and poorly defined [1].

Because of its rarity, there is limited evidence to guide optimal management strategies for angiosarcoma. In patients with localized disease who are suitable candidates for surgery, radical resection is the primary treatment option, whereas systemic therapy is the mainstay for unresectable or disseminated disease [2]. Weekly paclitaxel has prospective Phase II activity in unresectable angiosarcoma [16], and retrospective data suggest efficacy comparable to single‐agent doxorubicin in metastatic angiosarcoma [17]. Other reported systemic options include bevacizumab [18] and immune checkpoint inhibition [19]. In our patient, poor general condition (PS 3), ongoing bleeding, and advanced bilateral intrathoracic disease led to the choice of a relatively less intensive monotherapy approach with weekly paclitaxel. A recent case report has also described a partial response to paclitaxel in primary pulmonary epithelioid angiosarcoma [20].

In conclusion, this is a very rare case of thoracic epithelioid angiosarcoma presenting with bilateral hemothorax, hemoptysis, and histological evidence of alveolar bleeding. Although a pleural origin cannot be excluded with absolute certainty, the overall clinical, radiological, and operative findings favored pulmonary origin. The rapid progression and hemorrhagic presentation initially suggested infectious or autoimmune disease, but definitive diagnosis was achieved by surgical biopsy. Clinicians should maintain a high index of suspicion in patients who present with unexplained hemoptysis, recurrent bloody pleural drainage, ground‐glass opacities, and rapidly progressive nodular thoracic lesions, especially when more common infectious, autoimmune, thromboembolic, or neoplastic causes have been excluded.

## Funding

No funding was received for this manuscript.

## Conflicts of Interest

The authors declare no conflicts of interest.

## Data Availability

The data that support the findings of this study are available on request from the corresponding author. The data are not publicly available due to privacy or ethical restrictions.
